# Supplementary material for: Chlorhexidine Promotes Psl Expression in Pseudomonas aeruginosa That Enhances Cell Aggregation with Preserved Pathogenicity Demonstrates an Adaptation against Antiseptic
Source: Int J Mol Sci. 2022 Jul 27;23(15):8308. doi: 10.3390/ijms23158308 (PMC9368580; doi:10.3390/ijms23158308)
Supplement: Supplementary file 1 [file ijms-23-08308-s001.zip › ijms-1795213-supplementary.pdf]

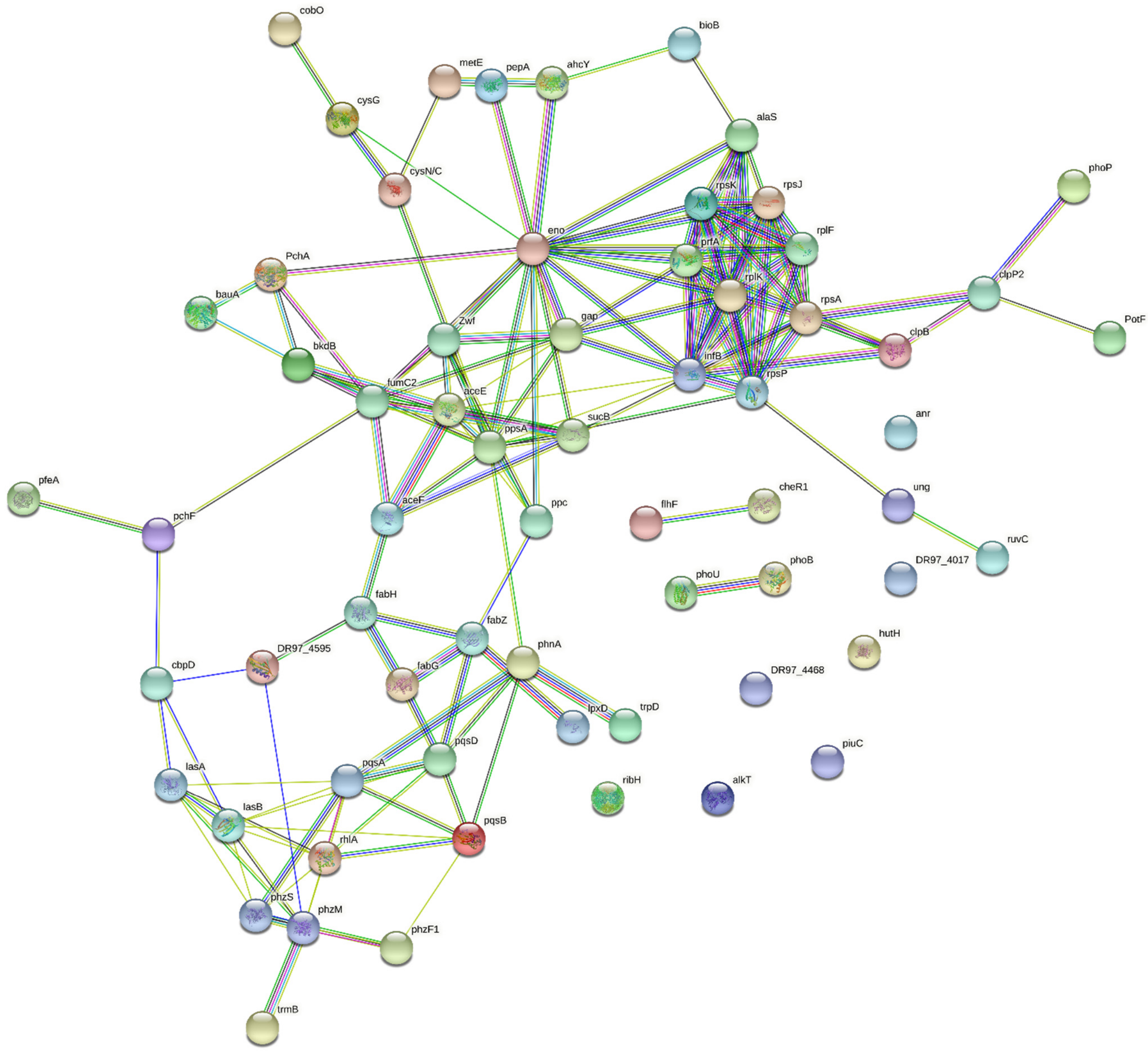

Supplement Figure S1. The protein association networks of downregulated proteins (p<0.01) from the proteomic analysis data.

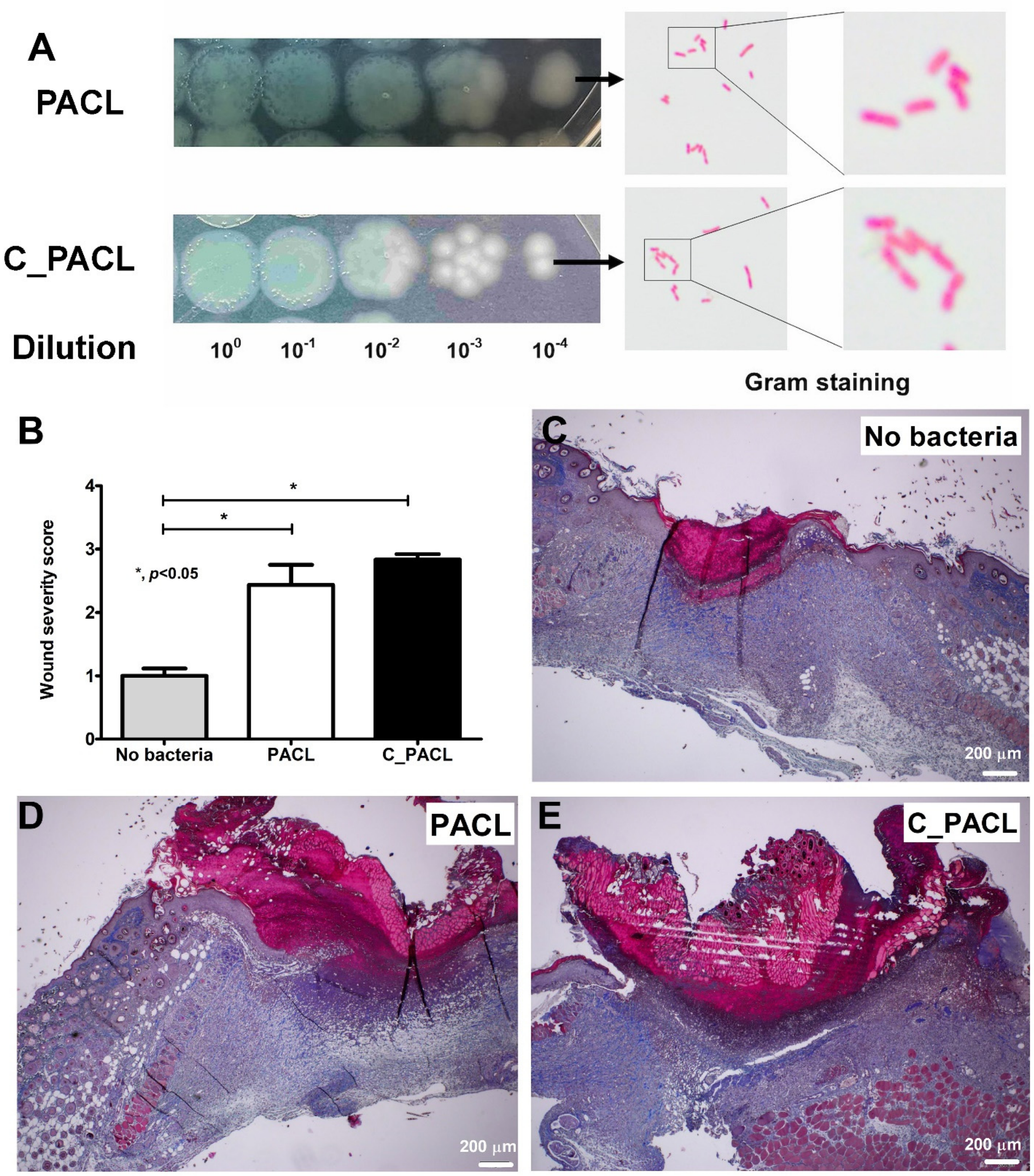

Supplement Figure S2. The colony morphologies after the 24 h incubation of wound fluid (the representative picture of figure 10C), using 5 µL of the wound fluid on the 3rd day of experiments diluted by normal saline solution (NSS) with the ratio between the wound fluid versus NSS at 1:1 to 1:10^4 (dilution 10^0 to 10^{-4}), in LB (Luria-Bertani) agar (the non-selective culture media) (A) demonstrates the larger colonies in the wound fluid from *P. aeruginosa* parent strain (PACL) compared with the Chlorhexidine (CHG)-treated strain (C_PACL) (the small variant colonies of C_PACL were visually separated) with the Gram-negative bacilli. Additionally, the characteristics of wound at the 14th day of experiments as indicated by histological scores (B) and the representative Hematoxylin

& Eosin (H&E) stained pictures from the wounds without or with bacterial administration tissues (C-E) are also demonstrated (n = 5-7 per group).

Supplement Table S1. Up-regulated proteins in *P. aeruginosa* biofilm after CHG treatment (*p*<0.001).

Protein ID	Gene name	Protein name	Fold change
Q02R89	*recA*	Protein RecA	1.594196974
Q9I2V0	*lpxH*	UDP-2,3-diacylglucosamine hydrolase	0.526893234
Q9HXJ8	*der*	GTPase Der	0.497557334
Q9HY64	*arnC*	Undecaprenyl-phosphate 4-deoxy-4-formamido-L-arabinose transferase	5.173517385
Q9HV35	*speH*	S-adenosylmethionine decarboxylase proenzyme	6.131500036
Q9HUA5	*opgG*	Glucans biosynthesis protein G	0.526388715
Q02TL9	*metK*	S-adenosylmethionine synthase	1.740803835
Q9HUW6	*ctpL*	Methyl-accepting chemotaxis protein CtpL	1.186734176
Q9HZF8	*pyrD*	Dihydroorotate dehydrogenase (quinone)	0.645713255
Q9HY63	*arnA*	Bifunctional polymyxin resistance protein ArnA	4.622647855
Q9HV32	*pmrA*	Response regulator protein PmrA	4.578510294
Q9HZE0	*gdhB*	NAD-specific glutamate dehydrogenase	0.951135173
Q9HV31	*pmrB*	Sensor protein kinase PmrB	4.587471354
Q9I6C8	*calB*	Probable coniferyl aldehyde dehydrogenase	4.022267089
P09591	*tufA*	Elongation factor Tu	0.12513888
Q9HY65	*arnB*	UDP-4-amino-4-deoxy-L-arabinose--oxoglutarate aminotransferase	3.892582755
Q51564	*dapF*	Diaminopimelate epimerase	0.404053594
Q02TE1	*gpFI*	Putative prophage major tail sheath protein	3.307847501
Q02G92	*rtcA*	RNA 3'-terminal phosphate cyclase	1.125912689
Q9I3N0	*ccmH*	Cytochrome c-type biogenesis protein CcmH	1.035865557
Q9HY61	*arnT*	Undecaprenyl phosphate-alpha-4-amino-4-deoxy-L-arabinose arabinosyl transferase	3.785571751
Q9I351	*folE2*	GTP cyclohydrolase 1 2	1.134229323

Supplement Table S2. Down-regulated proteins in *P. aeruginosa* biofilm after CHG treatment ($p<0.001$).

Protein ID	Gene name	Protein name	Fold change
Q9HXV3	*ppc*	Phosphoenolpyruvate carboxylase	-0.43186
Q9I618	*bioB*	Biotin synthase	-1.9266
Q9I1M0	*bkdB*	Lipoamide acyltransferase component of branched-chain alpha-keto acid dehydrogenase complex	-0.77054
Q9I4X2	*pqsB*	2-heptyl-4(1H)-quinolone synthase subunit PqsB	-0.70008
Q51404	*fumC2*	Fumarate hydratase class II 2	-6.44887
Q9HWH2	*phzM*	Phenazine-1-carboxylate N-methyltransferase	-2.29549
Q9HWG9	*phzS*	5-methylphenazine-1-carboxylate 1-monooxygenase	-1.59742
Q02L18	*lasA*	Protease LasA	-2.37045
Q9HXY6	*lpxD*	UDP-3-O-acylglucosamine N-acyltransferase	-0.90258
Q9HWF0	*rplF*	50S ribosomal protein L6	-0.46826
P0DPC1	*phzD2*	Phenazine biosynthesis protein PhzD2	-2.10236
Q9HXP9	*rpsP*	30S ribosomal protein S16	-0.61859
Q02TY0	*ahcY*	Adenosylhomocysteinase	-0.54656
Q9HYF3	*PA3453*	UPF0502 protein PA3453	-0.71213
Q51547	*phoU*	Phosphate-specific transport system accessory protein PhoU homolog	-1.1376
Q02I17	*trhO*	tRNA uridine (34) hydroxylase	-0.72178
P20582	*pqsD*	Anthraniloyl-CoA anthraniloyltransferase	-0.70176
Q05098	*pfeA*	Ferric enterobactin receptor	-0.92226
Q9HV55	*infB*	Translation initiation factor IF-2	-0.42999
Q9I4F9	*phoP*	Two-component response regulator PhoP	-0.51576
Q9I472	*cobO*	Corrinoid adenosyltransferase	-0.91966
Q9HWX5	*ribH*	6,7-dimethyl-8-ribityllumazine synthase	-0.77457
Q9HZ71	*rpsA*	30S ribosomal protein S1	-0.31819
O87131	*cheR1*	Chemotaxis protein methyltransferase 1	-0.83036
Q9I4X3	*pqsA*	Anthranilate--CoA ligase	-0.71889
O50274	*cysNC*	Bifunctional enzyme CysN/CysC	-0.6805
O68282	*zwf*	Glucose-6-phosphate 1-dehydrogenase	-1.40254
Q9HYR9	*clpP2*	ATP-dependent Clp protease proteolytic subunit 2	-1.47285
Q02T57	*rpsK*	30S ribosomal protein S11	-0.08474

Supplement Table S3. CHG adaptation in *P. aeruginosa* clinical isolates.

Strain	CHG MIC (mg/L)	CT MIC (mg/L)	Colony morphology	Strain	CHG MIC (mg/L)	CT MIC (mg/L)	Colony morphology
PA1	2.44	2	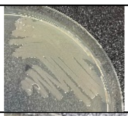	PA9	2.44	4[#]	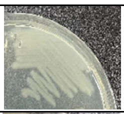
C_PA1	9.77	4[#]	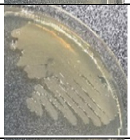	C_PA9	4.88	4[#]	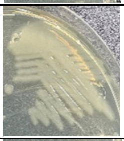
PA2	2.44	2	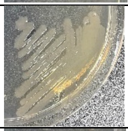	PA10	2.44	4[#]	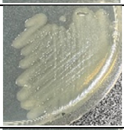
C_PA2*	4.88	2	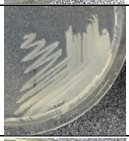	C_PA10*	>19.5	>16[#]	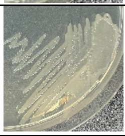
PA4	4.88	2	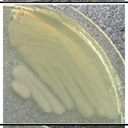	PA11	4.88	2	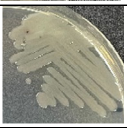
C_PA4*	19.5	16[#]	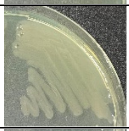	C_PA11	4.88	4[#]	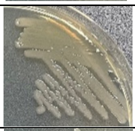
PA5	2.44	2	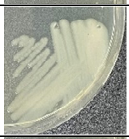	PA12	9.77	2	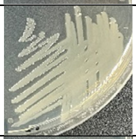
C_PA5*	4.88	8[#]	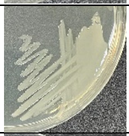	C_PA12*	9.77	4[#]	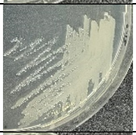
PA6	4.88	2	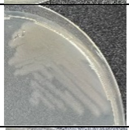	PA13	9.77	2	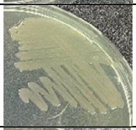
C_PA6*	4.88	16[#]	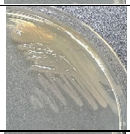	C_PA13*	9.77	4[#]	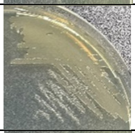
PA7	2.44	1	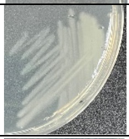	PA14	4.88	4[#]	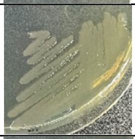
C_PA7*	4.88	2	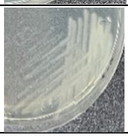	C_PA14	9.77	8[#]	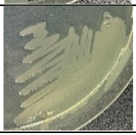

C_PA, CHG-adapted *P. aeruginosa*; *, small-colony variant; [#], colistin resistance (MIC≥4 mg/L)

Supplement Table S4. The minimal inhibitory concentration (MIC) breakpoints for *P. aeruginosa*.

Antimicrobial agent	Interpretation; MIC (mg/L)			Reference
	Susceptible	Intermediate	Resistant	
Chlorhexidine	ND	ND	ND	-
Colistin	-	≤2	≥4	[60]
Imipenem	≤2	4	≥8	[60]
Meropenem	≤2	4	≥8	[60]
Tobramycin	≤4	8	≥16	[60]

ND, No data available

Supplement Table S5. Oligonucleotide sequences of primers used for RT-PCR in this study.

**Primer name**	**Gene name**	**Oligonucleotide sequence (5′-3′)**	**Reference**
PA_algD_F	*algD*	GGGCTATGTCGGTGCAGTAT	[61]
PA_algD_R		AACGATACGTCGGAGTCCAG	
PA_pelA_F	*pelA*	AACGGATGGCTGAAGGTATG	This study
PA_pelA_R		ATCAAGCCCTATCCGTTCCT	
PA_pslB_F	*pslB*	GCGAGTTTCTCCTCAACACC	This study
PA_pslB_R		CGACCGTAGATGTCGTTGAA	
PA_siaA_F	*siaA*	ACTTCAATACCCTGCGCAAC	This study
PA_siaA_R		GATGAAGTCGTCGAGGAAGC	
PA_siaD_F	*siaD*	GCTACGCGAATACGACCTCT	This study
PA_siaD_R		ATTGACGGTCTGCGAATAGG	
PA_sadC_F	*sadC*	GTGGCCACCGACGAACTC	[47]
PA_sadC_R		TTAGGCACTGGTGACCTCCCA	
PA_gacS_F	*gacS*	CTGGGGATCATCAACGAGAT	This study
PA_gacS_R		ACCAGGTTGGTGAGGATCTG	
PA_gacA_F	*gacA*	CTATATCAGCCCGCAGATCG	This study
PA_gacA_R		CTTCTCGAAGATGCGGTAGC	
PA_ladS_F	*ladS*	GATCCGGCAGAACAACTAC	This study
PA_ladS_R		GGACGCTGAGGAAGATGAAC	
PA_rsmY_F	*rsmY*	AGGACATTGCGCAGGAAG	This study
PA_rsmY_R		GGGGTTTTGCAGACCTCTATC	
PA_rsmZ_F	*rsmZ*	CGTACAGGGAACACGCAAC	This study
PA_rsmZ_R		TATTACCCCGCCCACTCTTC	
PA_rsmA_F	*rsmA*	GGACCTGATCCCTCTGTTGA	This study
PA_rsmA_R		GAAGTGCATGTCCTCGATCA	
PA_pilA_F	*pilA*	GCTTCTACTGCGACCGAAAC	This study
PA_pilA_R		CGCAGTACGGTTCAGAGTGA	
PA_oprF_F	*oprF*	GGTTACTTCCTGACCGACGA	This study
PA_oprF_R		TCGCTGTTGATGTTGGTGAT	
PA_16S_rRNA_F	*16S rRNA*	ACGCAACTGACGAGTGTGAC	[61]
PA_16S_rRNA_R		GATCGCGACACCGAACTAAT	
Human_TLR-2_F	*TLR-2*	TCCTCCAATCAGGCTTCTCTGTCTT	[63]
Human_TLR-2_R		CTCGCAGTTCCAAACATTCC	
Human_TLR-4_F	*TLR-4*	CACAGACTTGCGGGTTCTAC	[63]
Human_TLR-4_R		AGGACCGACACACCAATGATG	
Human_TLR-5_F	*TLR-5*	AGCTTCAACTATATCAGGACA	This study
Human_TLR-5_R		TGGTTGGAGGAAAAATCTAT	
Human_TLR-6_F	*TLR-6*	GGCCCTGCCCATCTGTAAGG	[63]
Human_TLR-6_R		ACTCTCAACCCAAGTGCAGT	
Human_TNF-α_F	*TNF-α*	CCTCACACTCAGATCATCTTCTC	[61]
Human_TNF-α_R		AGATCCATGCCGTTGGCCAG	
Human_IL-6_F	*IL-6*	ATGAACTCCTTCTCCACAAGC	[63]
Human_IL-6_R		GTTTTCTGCCAGTGCCTCTTTG	
Human_IL-8_F	*IL-8*	CTGTGAGTTATGCGCCGAAGA	This study
Human_IL-8_R		TGGTGCTGTACATTGGGGTTG	
Human_TGF-β_F	*TGF-β*	CAGAGCTGCGCTTGCAGAG	[61]
Human_TGF-β_R		GTCAGCAGCCGGTTACCAAG	
Human_GM-CSF_F	*GM-CSF*	CACTGTGGTCTGCAGCATCT	This study
Human_GM-CSF_R		AGGTGAGTCTGCAGGCATTT	
Human_iNOS_F	*iNOS*	ACCCACATCTGGCAGAATGAG-	[61]
Human_iNOS_R		AGCCATGACCTTTCGCATTAG	
Human_Arg-1_F	*Arg-1*	CTTGGCTTGCTTCGGAACTC	[61]
Human_Arg-1_R		GGAGAAGGCGTTTGCTTAGTTC	
Human_IL-10_F	*IL-10*	TCTCCGAGATGCCTTCAGCAGA	[61]
Human_IL-10_R		TCAGACAAGGCTTGGCAACCCA	
Human_β-actin_F	*B-actin*	CGGTTCCGATGCCCTGAGGCTCTT	[61]
Human_β-actin_R		CGTCACACTTCATGATGGAATTGA	

References

47. Zhu, B.; Liu, C.; Liu, S.; Cong, H.; Chen, Y.; Gu, L.; Ma, L.Z. Membrane association of SadC enhances its diguanylate cyclase activity to control exopolysaccharides synthesis and biofilm formation in *Pseudomonas aeruginosa*. *Environ Microbiol* **2016**, *18*, 3440-3452, doi:10.1111/1462-2920.13263.
60. CLSI. *Performance Standards for Antimicrobial Susceptibility Testing*, 30th ed.; Clinical and Laboratory Standards Institute: Wayne, PA, USA, 2020.
61. Phuengmaung, P.; Somparn, P.; Panpetch, W.; Singkham-In, U.; Wannigama, D.L.; Chatsuwan, T.; Leelahavanichkul, A. Coexistence of *Pseudomonas aeruginosa* With *Candida albicans* Enhances Biofilm Thickness Through Alginate-Related Extracellular Matrix but Is Attenuated by *N*-acetyl-l-cysteine. *Front Cell Infect Microbiol* **2020**, *10*, 594336, doi:10.3389/fcimb.2020.594336.
63. Phuengmaung, P.; Panpetch, W.; Singkham-In, U.; Chatsuwan, T.; Chirathaworn, C.; Leelahavanichkul, A. Presence of *Candida tropicalis* on *Staphylococcus epidermidis* Biofilms Facilitated Biofilm Production and *Candida* Dissemination: An Impact of Fungi on Bacterial Biofilms. *Front Cell Infect Microbiol* **2021**, *11*, 763239, doi:10.3389/fcimb.2021.763239.
